# Anatomic and refractional correlations in earliest glaucomatous visual feld defects

**DOI:** 10.5935/0004-2749.2021-0042

**Published:** 2022-09-06

**Authors:** Alexis Galeno Matos, João Augusto Lima Bisneto, Felipe Moraes Lopes, Hermano Queiroz Gurgel, Magno Martins Pinto de Faria

**Affiliations:** 1 Fundação Leiria de Andrade, Fortaleza, CE, Brazil; 2 Centro Universitário Christus - Unichristus, Fortaleza, CE, Brazil

**Keywords:** Glaucoma, Ametropia, Foveal disk angle, Scotoma, Glaucoma, Ametropia, Ângulo disco fóvea, Escotoma

## Abstract

**Purpose:**

In glaucoma, initial visual field scotomas can be peripheral or central, whereas central scotomas are more severe and can disrupt daily activities. Individual anatomical features may influence the distribution of retinal nerve fibers and the starting site of visual field defects in glaucoma. In this study, we aimed to correlate myopia and hyperopia or anatomical variation of the disk-fovea angle with initial central or peripheral lesions in the visual field.

**Methods:**

This cross-sectional study included patients with primary open-angle glaucoma divided into a group of isolated central or peripheral scotomas in the visual field with MD > or equal to -6 dB, correlating with the degree of ametropia and anatomical variations, such as the disk-fovea angle.

**Results:**

We included 52 patients with glaucoma. Of 20 myopic patients, 6 (30%) had central scotomas, and 14 (70%) had peripheral scotomas. Of 32 hyperopic patients, 12 (37.5%) had central scotomas, and 20 (63.5%) had peripheral scotomas. Regarding the disk-fovea angle, 25 eyes had the disk-fovea angle of < -7°, with 9 (36%) eyes presenting with central scotoma, and 27 eyes presented with the disk-fovea angle of > -7°, with 9 (33.3%) eyes presenting with a central scotoma.

**Conclusion:**

This study showed an association between ametropia and scotomas on the perimetry in patients with glaucoma. Patients had a higher incidence of peripheral scotomas, but hyperopic patients had a greater number of central scotomas than myopic patients, and myopic patients had more peripheral scotomas than hyperopic patients. The disk-fovea angle was not correlated with scotomas in initial glaucoma.

## Abstract (pt)

**Objetivos:**

No glaucoma, os escotomas iniciais no campo visual podem ser periféricos ou centrais. Os escotomas centrais são mais graves e podem atrapalhar as atividades diárias. As características anatômicas individuais podem influenciar a distribuição das fibras nervosas da retina e o local inicial dos defeitos do campo visual no glaucoma. Nesse estudo buscamos correlacionar a miopia e a hipermetropia ou a variação anatômica do ângulo disco-fóvea com lesões iniciais centrais ou periféricos no campo visual.

**Métodos:**

Este foi um estudo transversal incluindo pacientes com glaucoma primário de ângulo aberto divididos em grupo de escotomas isolados central ou periférico no campo visual com MD > ou igual a -6 dB, correlacionando com o grau de ametropia e variações anatômicas como o ângulo disco fóvea.

**Resultados:**

Incluímos 52 pacientes com glaucoma. Dos 20 míopes, 6 (30%) apresentaram escotoma central e 14 (70%) escotoma periférico) e 32 olhos hipermetrópicos com 12 (37,5%) escotomas centrais e 20 (63,5%) escotomas periféricos. Sobre o ângulo disco fóvea, 25 olhos tiveram ângulo disco fóvea menor que -7º com 9 (36%) olhos apresentando escotoma central e 27 olhos com ângulo disco fóvea maior que -7º com 9 (33,3%) olhos apresentando escotoma central.

**Conclusão:**

Este estudo mostrou associação da ametropia e os escotomas na perimetria em pacientes com glaucoma. Pacientes apresentaram maior incidência de escotomas periféricos, porém hipermétropes apresentaram maior quantidade de escotoma central que os míopes e os míopes mais escotomas periféricos que os hipermetropes. O ângulo disco fóvea não foi correlacionado com escotomas na perimetria no glaucoma inicial.

## INTRODUCTION

Glaucoma is one of the leading causes of visual loss. Glaucoma is characterized by progressive neuropathy resulting in located defects in the visual field. However, the initial defects and mode of disease progression vary greatly^(1,2)^. Visual field defects in patients with glaucoma are ultimately explained by damage to the retinal ganglion cells (RGCs) and can be detected by the anatomical path of the axons in the retinal nerve fiber layer (RNFL)^(3)^. Standard automated perimetry (SAP) is the gold standard to detect and monitor visual loss in patients with glaucoma^(4)^.

The concept that a defect in the visual field always starts at the periphery, with the relative preservation of the central field, in glaucoma patients is incorrect^(5)^. A study by De Moraes et al. showed that the initial defects can be peripheral or central^(5^).

 Although the peripheral nasal step is the most common defect, superior para-central scotoma is the second most prevalent defect^(6)^. Defects, even small initial ones, affecting central fixation tend to have a greater impact on the patient’s visual function^(1)^.

Evidence shows that 70% of the initial defects on perimetry are in one quadrant, and in 56% of the cases, they continue to evolve in the same quadrant^(7)^. Also, systemic and refractive anatomical variations, mostly of the posterior pole, directly influence the initial location of the visual field defect^(8)^. The anatomical position of the fovea and its relationship between distance and angulation to the optic nerve can affect macular RNFL distribution^(9)^ and the disk-fovea angle (DFA)^(10,11)^, as the axial length (LA) promotes increased risk of central visual field lesions^(12)^.

Research has shown that patients diagnosed with ametropia could more frequently have anatomical changes in the posterior ocular pole and may have some association with glaucoma or behave as a bias in their field evaluation based on optical coherence tomography (OCT)^(13)^. Therefore, we correlated the anatomical variations, the spherical diopter equivalent, and initial visual field defects in patients with early glaucoma based on the above-mentioned concepts and the lack of studies on the correlation of ametropia with interference in the initial defects.

## METHODS

### Participants

This was a cross-sectional study based on the survey of patients’ medical records in the glaucoma department of Hospital das Clinicas, Ribeirão Preto Medical School, University of São Paulo and Hospital de Olhos Leiria de Andrade. Data on visual acuity with the more recent recorded refractive correction, slit-lamp biomicroscopy, measurement of intraocular pressure (IOP) (Goldmann tonometer), gonioscopy, fundus examination, and disk retinography with Topcon TRC 50 were collected in the evaluation protocol. DX (Topcon Corporation, Tokyo, Japan). SAP automatic perimetries were also surveyed using the 24-2 SITA Standart - Humphrey (Carl Zeiss Meditec Inc., Dublin, CA). The study protocol was approved by the Ethics Committees and adhered to the principles of the Declaration of Helsinki.

Inclusion criteria comprised phakic patients with primary open-angle glaucoma diagnosed based on the presence of reproducible changes in SAP with corresponding evidence of glaucomatous neuropathy in at least one eye. The altered result was defined as a standard deviation (SD) with a p-value of <0.05 and/or GHT results outside normal limits. Only reliable tests were included (< 20% of fixation loss, false negatives, and <20% of false positives). Patients with ptosis, coexisting retinal disease, dense cataract with visual acuity worse than 20/60 (0.33 Snellen or 0.48 LogMAR), spherical equivalent refraction (SER) > ± 6.0 D, or axial length of ≤22 and ≥26 mm eon optical biometrics, and perimetry with MD <than -6 dB were excluded from the study.

### Groups

**Figure 1:**
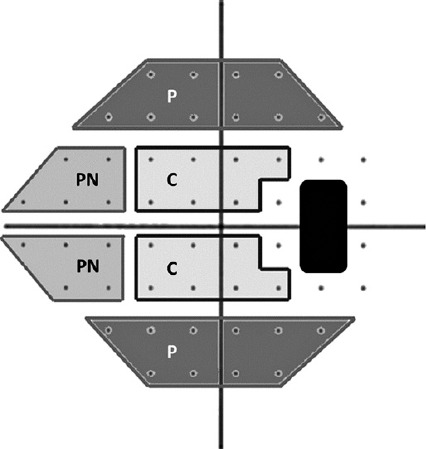
Delimitation of the proposed sectors (shadow areas) in the graph of the pattern deviation 24-2. Peripheral (PN: nasal peripheral and P: peripheral) and central (C) sectors in the superior or inferior hemicampus.

Patients were divided into two groups according to the spherical diopter equivalent: hyperopia with positive SER and myopia with negative SER. The defects in the perimetry were allocated in 3 sectors of the Pattern deviation graph: upper or lower central defect (C), peripheral superior or inferior (P), and peripheral nasal defect (PN) superior or inferior (Figure 1). Additionally, the peripheral injury extending across the two sectors in the same hemicampus, excluding central or peripheral defects, was included. We followed Anderson’s criteria to define glaucomatous visual field defect (at least 3 points with a p-value of <5%, of which at least 1 has a p-value <1%) with MD up to -6 dB.

The patients were further classified into two other groups based on the DFA. A group was formed by patients with DFA of < -7°, and another group was formed by patients with DFA of > -7°. Negative angulation below the midline joining the center of the optic nerve and the macula was considered. We used -7° as the division between groups as this is the average DFA found in studies^(11,14)^.

### Retinography analysis

The images were acquired using a retinograph with a 45° angle field. Patients underwent the exam after pupil dilation. All images were evaluated using ImageJ software (http://rsb.info.nih.gov/ij/index.html) from the “National Institutes of Health, Bethesda, MD”. Two different masked evaluators (A.G.M and J.A.L.B, both with experience in ophthalmology and software use) performed the analysis of the foveal disk angle. Interob-server agreement between the evaluators was assessed using an intraclass correlation coefficient (ICC). This coefficient can take a value from 0 to 1, with 0 indicating no agreement and 1 indicating perfect agreement. We calculated the value of the average angle between the measurements of the 2 evaluators if ICC reached an appropriate value.

### Sample size and statistical analysis

The sample size calculation was based on the SD of the DFA measurements from previous studies^(10,14,15)^. Considering an α value of 0.05 and a study power of 90%, at least 20 eyes per group should have been analyzed. Descriptive statistics included mean, SD, and t-test for normally distributed variables and median and interquartile range. For categorical variables, we used Fisher’s exact test and a unilateral analysis of variance. All statistical analyzes were performed with STATA, version 13 (StataCorp LP, College Estação, TX).

## RESULTS

A total of 52 eyes was included in the study, of which 20 had myopic SER and 32 had hyperopic SER. Most patients were female (36 eyes), and the average age was 59 years ± 10.81. Still, the patients in the myopia group were younger than those in the hyperopia group (p=0.0087) (Table 1).

**Table 1:** Demographic and clinical data of the patients included in the study divided according to the refraction

	Myopia (n=20)	Hyperopia (n=32)	p-value
Gender (M/F)	8M/12F	8M/24F	
Age (years)	54.9 ± 10.9	61.9 ± 9.94	0.0087
Spherical equivalent refraction (SER)	1.4 ± 1.5	2.27 ± 1.05	0.0069
MD (- / dB)	3.31 ± 1.9	2.78 ± 1.57	0.1379
Pachymetry (µm)	503 ± 20	505 ± 40	0.4207
VFI (%)	95%	93%	
Average DFA (º)	7.87 ± 3.9	7.5 ± 3.1	0.3557
Hemicampus of scotoma			
Superior (%)	12 (60)	23 (28.1)	0.0197
Inferior (%)	8 (40)	9 (71.9)	0.0485
Scotoma local			
Central (%)	6 (30)	12 (37.5)	0.0162
Peripheral (%)	14 (70)	20(62.5)	0.0011
Nasal periphery (PN)	2	7	
Peripheral (P)	9	10	
Peripheral + Nasal peripheral	3	3	

SER= spherical equivalent refraction; MD= mean deviation; VFI= visual field index; DFA= disk-fovea angle; NP= nasal periphery; P= peripheral.

We found an ICC of 0.87 (95% confidence interval) for the graduation agreement for masked angle measurements. The average DFA was -7.66° ± 3.40°. The superior hemicampus had more scotomas (central or peripheral) compared to the inferior hemicampus (35 vs. 17). When we compared the central scotomas, we found only 4 eyes that had inferior central scotoma compared to 14 that had superior central scotoma.

There were no significant differences between laterality (right or left) within the groups. Variables, such as mean deviation (MD) and VFI of the visual field, SER, and central corneal thickness, were not statistically different between the groups. Both groups, the myopic and hyperopic ones, had a higher number of peripheral defects; however, the myopic group had 30% of central defects compared to 37.5% in the hyperopia (p=0.0162) (Table 1).

All eyes presented a negative inclination of the foveal disk angle (myopia: -7.87° ± 3.9° and hyperopia: -7.5° ± 3.1°). The analysis was classified according to Table 2 when scotoma evaluation in the perimetry was correlated to the DFA measurement, without statistical difference between the scotoma sectors of the visual field.

**Table 2:** Clinical data of the patients included in the study divided according to the foveal disk angle

	DFA<-7º (N=25)	DFA>-7º (N=27)	p-value
Average DFA (º)	4.95 ± 1.49	10.18 ± 2.65	0.0001
Hemicampus of scotoma			
Superior (%)	16 (64)	19 (70.3)	0.33
Inferior (%)	9 (36)	8 (29.7)	0.3745
Scotoma local			
Central (%)	9 (36)	9 (33.3)	0.3557
Peripheral (%)	16 (64)	18 (77.7)	0.3050
Nasal periphery (PN)	4	5	
Peripheral (P)	10	9	
Peripheral + Nasal Peripheral	2	4	

DFA= disk-fovea angle; NP= nasal periphery; P= peripheral.

## DISCUSSION

Currently, we cannot predict what type of visual field defect the patient will develop, whether it be a nasal step or a central scotoma. There is a wide variation in the thickness and normal distribution of RNFL, being affected by age, ethnicity, axial length, area of the optic disk, and the relative position of the fovea^(13)^. Refractive errors are usually associated with the location of the main bundles of the RNFL in addition to the axial length^(16)^.

Central scotomas can impact more intensely on daily activities and should be treated with greater rigor^(11)^. In our research, we had a greater number of scotomas in the superior hemicampus compared to the inferior hemicampus, especially when referring to central scotomas (4 inferior vs. 14 superior). Hood et al. developed the concept of a macular vulnerability zone as the region between the lower portion of the temporal quadrant and the temporal portion of the lower quadrant of the optic disk. Within this zone, the RGC axons of the lower macular region would be at greater risk of damage compared to those of the upper macula entering the temporal quadrant^(17)^.

In our study, the age difference between the myopic (54.9 ± 10.9 years) and hyperopic (61.9 ± 9.94 years) groups could influence the global sensitivity; however, our analysis was based on the pattern deviation scotomas. There were more women in our study compared to men (36 vs. 16), but it was not associated with a greater tendency to paracentral findings, unlike the finding by Kim et al. that showed patients with paracentral scotoma were predominantly female with a higher incidence of disk hemorrhages^(1)^.

Studies suggest that a longer axial length changes the disposition of the median raphe, compressing the temporal fibers and lengthening the nasal fibers^(18)^ myopic eyes have more “curvy” than emmetropic or hyperopic, which implies a steeper inclination to the nasal optic nerve head^(19,20)^. High axial myopia is a risk factor for glaucoma development and deterioration. Structural changes associated with myopia, such as a longer axial length, a larger or tilted optic disk, and peripapillary atrophy (PPA), can make the macular papillary bundle more susceptible to glaucoma^(1^).

**Figure 2:**
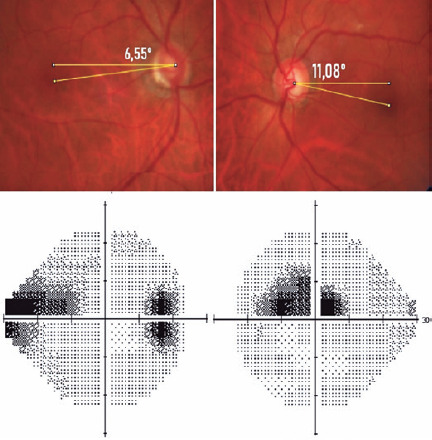
Patient (right eye: -2.00 SR and left eye: +2.25 SR) with inferior temporal notch in both eyes and in the perimeter of the right eye (DFA: -6.55°, AL: 25.67 mm) peripheral nasal scotoma and left eye (DFA: -11.08°, AL: 23.64 mm) central scotoma.

In our study, we only used the axial length of ≤22 and ≥26 mm, and the differences between the groups were significant (24.32 ± 0.8 mm in the myopic group vs. 23.78 ± 0.6 mm in the hyperopic group). We demonstrated that the myopic group had a higher prevalence of peripheral scotoma compared to central scotoma (central 30% vs. peripheral 70%) and differed from the hyperopic group, which had more central lesions (37.5% central vs. 62.5% peripheral). Changes in nerve fiber distribution and inclination of the optic disk in myopic eyes may predispose eyes to more peripheral arcuate glaucomatous damage^(21)^. Our findings differ from those of Jung et al., who reported that myopic patients were more likely to have initial paracentral scotoma compared to peripheral scotoma^(22)^. In Figure 2 of the same patient with a myopic right eye and hyperopic left eye presenting with an inferior temporal notch in both eyes, defects in the visual field illustrate our results, i.e., the tendency to develop peripheral scotoma in myopic eyes. Comparing the groups, we verified with a statistical difference that the hyperopic group had more central scotomas compared to patients in the myopic Group (6 vs. 12, p=0.0162).

In our assessment, the average DFA found (-7.66° ± 3.40°) was higher than that found by other authors^(11,14)^, but between the evaluated groups (myopic vs. hyperopic), the DFA had no statistical difference. Also, comparing the groups with the highest and lowest DFA (Table 2), there was no significant association with the initial defect location. In some studies, the DFA variation might have influenced a higher prevalence of central defects^(11,21)^. We justify this difference by the small number of patients in the study, a different number of participants between the groups (20 eyes in the myopic Group vs. 32 eyes in the hyperopic Group), and all patients having negative DFA. Matos et al. did not report an association of the central defect with DFA but with the vertical deviation of the fovea^(10)^.

One of the main limitations of this study was the possibility of inclination and rotation of the optic nerve. Despite this, the present study excluded patients with high myopia or hyperopia based on the equivalent external spherical refraction of > ± 6.0 D or axial length of ≤22 and ≥26 mm in optical biometric measurements. Thus, it minimized the chance of having disk anomalies with a significant inclination and/or rotation in our sample. We would also like to highlight that the refraction could be influenced by the early opacification of the lens, although we used the most recent refraction recorded in the chart, and all patients had visual acuity of better than 20/60 (0.33 Snellen or 0.48 LogMAR) in the eye included in the study. We performed DFA measurements in a masked way and with good agreement between measurements, although the measurements were examiner-dependent. The sample size calculation was based on previous studies^(10,14,15)^, and the low number of participants can also be a limitation of the study.

In conclusion, our results suggest that individual characteristics, such as low myopia or hypermetropia, can influence the initial location of visual field defects in glaucoma. The superior hemicampus was more vulnerable to defects, especially central-region defects. Both groups had a prevalence of peripheral defects, but eyes with a myopic equivalent degree had a greater tendency to peripheral defects than central, and hyperopic eyes had more central scotomas compared to myopic eyes. The DFA was not decisive in predicting the location of the visual field defect. The interpersonal and anatomical variations peculiar to each individual must be analyzed when prescribing treatment; hence, individualized.
